# Adapting routines in schools when facing challenging situations: Extending previous theories on routines by considering theories on self-regulated and collectively regulated learning

**DOI:** 10.1007/s10833-022-09459-1

**Published:** 2022-07-07

**Authors:** Katharina Maag Merki, Andrea Wullschleger, Beat Rechsteiner

**Affiliations:** Institute of Education, University of Zurich, Freiestrasse 36, 8032 Zurich, Switzerland

**Keywords:** Adapting routines in schools, Challenging situation, Educational change, Self-regulated and collectively regulated learning, Theoretical framework

## Abstract

Routines play a major role in educational change in schools. But what happens if the routines performed by school staff fail to deal successfully with current challenges? What strategies aid adaptation of the routines in a specific situation? Up to now, there exists no comprehensive concept for understanding why and at what points the adapting of routines in schools in a specific situation takes a favorable or unfavorable direction. To address this gap, we propose extending theories on routines by considering theories on self-regulated and collectively regulated learning. We consider these theories to be a beneficial complement because of their broad theoretical, methodological, and empirical research base. We argue that these theories enhance the understanding of adapting routines to specific challenging situations in schools. We present a newly developed theoretical framework for dealing with specific challenging situations in schools as an interplay between routines and regulation processes. Finally, important research questions regarding the suggested approach are discussed.

## Introduction

Routines in schools play a major role in educational change (Coburn & Turner, 2011; Horn & Little, 2010; Spillane et al., 2011; Tubin, 2015). Not only do routines structure day-to-day working processes and support the organization’s performance when time is short, but they are also essential when dealing with challenging situations. But what happens if the routines of teachers, school leaders, or school teams fail to deal successfully with a concrete challenging situation? What strategies help teachers and school teams adapt dysfunctional routines so that they can, without delay, improve the fit between what they normally do and what needs to be done in the face of a challenging situation?

Several studies have shown how routines might change over a longer term (Conley & Enomoto, 2005; Hatch & Hill, 2016; Sherer & Spillane, 2011; Spain, 2017; Spillane et al., 2016). However, there is a lack of studies that can provide insights on how to adapt routines without delay and effectively to overcome a specific challenging situation. Feldman (2000) discussed this aspect in terms of “repairing the routine” (p. 620). Repairing routines is one strategy used to change routines intentionally in a specific situation to deal competently with the concrete challenge. Repairing routines helps actors reduce unintended and undesirable outcomes and produce the intended outcome by increasing the “quality of thinking” (Earl & Louis, 2013, p. 202) or the “depth of inquiry” (Schildkamp et al., 2016, p. 232). However, few studies have provided insights on what this repairing process looks like and how routines can be effectively adapted to meet specific goals when dealing with a concrete challenging situation in schools. Accordingly, adapting routines in a specific challenging situation can only be described insufficiently, and there is a lack of concepts for understanding why and at what points the adaptation of routines takes a favorable or unfavorable course.

To close this gap, we propose extending previous theories on routines by introducing *theories on self-regulated and collectively regulated learning as a new theoretical lens.* We consider these theories to be a beneficial complement because of their broad theoretical, methodological, and empirical research base (Hadwin et al., 2018; Panadero, 2017; Panadero & Järvelä, 2015). Most importantly, theories on regulated learning are framed by a socio-constructivist understanding of learning (Vygotsky, 1978) and will aid identification of the conditions and analysis of teachers’ and school teams’ adaptation of routines in a concrete challenging situation by applying cognitive, metacognitive, and motivational regulation strategies (Winne & Hadwin, 1998, 2008). Further, theories on regulated learning analyze not only individual but also collective learning processes (Bakhtiar & Hadwin, 2020; Hadwin et al., 2011, 2018; Panadero & Järvelä, 2015). This is most important, as schools, as loosely coupled systems (Weick, 1976), have a highly complex social structure, and the handling of challenging situations is undertaken not only by individual teachers but also by school teams or the whole school (Mitchell & Sackney, 2011; Stoll & Louis, 2007).

Initial studies have looked at some dimensions of regulated learning in the context of teachers’ professional learning, self-evaluation, and data use (Butler et al., 2004; Kwakman, 2003; Muijs et al., 2014; Persico et al., 2015; Schildkamp et al., 2016; Sitzmann & Ely, 2011). However, so far there has been no discussion of theories of regulated learning that analyzes routines of teachers, school leaders, or school teams and the need to adapt routines when facing a specific challenging situation in everyday school life.

The aim of this paper is therefore to elaborate on this new theoretical approach and extend the existing research on adapting routines in schools. To this end, we first present previous theoretical and empirical research on routines and how they can be adapted and changed. We go on to describe the research gap and how we plan to address it. As a further basis, we present the core elements of regulated learning theories. We then present the newly developed theoretical framework of adapting routines. Finally, important research questions in relation to the suggested approach are discussed.

## Routines in schools: Conceptual clarifications and previous research

### Routines in schools

An organizational routine is a “repetitive, recognizable pattern of interdependent actions, involving multiple actors” (Feldman & Pentland, 2003, p. 95). By applying routines actors can take action in a prescribed way. Routines are crucial to ensure an organization’s performance even when time, attention, information, and certainty may be lacking (Conley & Enomoto, 2005; Levitt & March, 1988; March & Olsen, 1975; Miner et al., 2008).

Feldman and Pentland (2003) differentiated two aspects: routines as standard procedures (ostensive aspect), or as Becker (2004) calls it, ‘if–then rules,’ and the actual performance of routines in a concrete situation (performative aspect). Sometimes the ostensive aspect is associated with artifacts, which include elements such as checklists, scripts, or formal procedures (Feldman & Pentland, 2003; Pentland & Feldman, 2005). According to Feldman and Pentland (2003), referring to Giddens’ (1984) structuration theory, the ostensive and performative aspects of organizational routines are recursively related, “with the performances creating and recreating the ostensive aspect and the ostensive aspect constraining and enabling the performances” (p. 105).

Although these theories were not developed within educational research, educational researchers have used these concepts to better understand educational change in schools (Coburn & Russell, 2008; Coburn & Turner, 2011; Enomoto & Conley, 2008; Hatch & Hill, 2016; Horn & Little, 2010; Kerrigan, 2015; Park et al., 2017; Spillane et al., 2011, 2016; Tubin, 2015; Tyack & Tobin, 1994). Researchers have identified several routines in relation to teaching and learning, although the concepts differ. Spillane et al. (2016) identified professional learning communities as organizational routines in schools that structure teachers’ discussions. Hatch and Hill (2016) characterized instructional rounds as organizational routines, and Tubin (2015) found several routinized processes that lead to high achievements, such as developing a vision or building a senior leadership team.

Additionally, as educational change processes in schools are related to data-based learning processes, routines in data use can be identified (Coburn & Turner, 2011; Spillane, 2012). Coburn and Turner defined routines for data use “as the modal ways that people interact with data and each other in the course of their ongoing work” (2011, p. 181). In line with Feldman and Pentland (2003), they understand a data use routine as a “recurrent and patterned interaction that guides how people engage with each other and data in the course of their work” (p. 181). Coburn and Turner assumed that data use routines might influence the interpretive process in data use and what people notice and discuss. Accordingly, “they can alternatively open up or close down opportunities for learning” (p. 182). According to Spillane (2012), organizational routines are a useful unit of analysis for studying data use because they help to analyze “standard ways of doing things in the school and how, if at all, these standard ways of doing things change in response to data-use initiatives” (p. 117).

### How routines can be changed

In the literature, changing routines refers to both long-term and situation-specific short-term adaptations or, with reference to Feldman and Pentland (2003), to the ostensive and performative aspect of routines. Many studies found that it is difficult to change routines, particularly if they are strongly embedded in organizational structures (Feldman, 2003; Feldman & Pentland, 2003; Howard-Grenville, 2005; Rice & Cooper, 2010). Still, there is empirical evidence that under certain conditions and in specific situations, routines can be changed. Previous studies, although only few are available for the school context, have identified a variety of strategies; the research has focused on strategies that intentionally change routines to create learning either by: (a) exogenous forces when the context is restructured (e.g., change of policy, rules, responsibilities), or (b) endogenous forces in the process of interaction by continuously performing routines within the organization.

In the following, we provide a brief overview of studies that examined strategies for changing routines. However, in the studies a clear differentiation between adapting a routine to a concrete specific situation (performative aspect) and changing routines in a longer perspective (ostensive aspect), is not sufficiently visible. Accordingly, the literature review can only give a general overview on how routines might be changed.

Feldman (2000) described three strategies for changing routines in organizations: repairing routines, expanding routines, and striving for improvements in the routines. Repairing has the function of restoring the routine to a stable balance but is not directly associated with long-term change. By expanding the routines and striving for improved routines, long-term changes can be implemented, such as adding new elements to the routine, implementing new rules, changing policy, or changing the roles and responsibilities of central administrators. Another long-term change strategy is described by Fiol and O’Connor (2017a, b) as the unlearning of established routines to implement new and more effective routines. Fiol and O’Connor argued that routines can be changed by destabilization, meaning questioning old routines (e.g., by external environmental forces, or internal failures as disruptions that may trigger initial destabilization of old routines), discarding as a process of letting go, and experimenting as a process of learning new routines. A reflective process to change routines is also described by Rice and Cooper (2010). They argue that to resolve and mitigate “unusual routines” that create unintended and undesirable outcomes, organizations need the ability to identify the existence of an unusual routine and to change them by implementing feedback systems, e.g., through walkarounds.

Rerup and Feldman (2011) followed another argumentation, drawing on cognitive learning theories. They investigated how processes of trial-and-error learning relate actions to the ostensive aspect of routines and to enacted organizational schemata. Two other studies, also related to cognitive learning theories, examined learning from the experience of others: Kim and Miner (2007) analyzed conditions under which learning from failures take place, and Bresman (2013) examined how groups change routines drawing on the experience of other groups, through the subprocesses of identification (a group identifies another group with prior related experiences), translation (a group translates knowledge developed by another group to reach a judgment about its value), adoption (a group embeds knowledge developed by another group in an existing routine, resulting in a changed routine), and continuation (subprocess that determines whether a group continues to rely on a changed routine).

In contrast, Rerup (2009) focused on strategies to prevent an organizational crisis from reoccurring through a process of focusing attention on salient and non-salient cues in organizations at the same time. Organizations have to increase their ability to notice, encode, interpret, and focus on important issues that may not yet be the focus of their attention and are not yet represented by implemented routines (Rerup, 2009).

In the school context, Conley and Enomoto (2005, 2009) identified factors that stimulate changes in routines, such as increased communication and connections to gain a shared understanding, more comprehensive data management, more accurate reporting, change of resources by the management, reinforcing and establishing power arrangements, and establishing new possibilities in terms of roles and new responsibilities for individuals (e.g., shifting roles from the administrators to the teachers). In the context of data use, Coburn and Turner (2011) found that tools (e.g., protocols for data analysis, formative assessment systems), comprehensive data initiatives, and high-profile policy initiatives that promote data use are important. These designed routines have the potential “to (a) shape what teachers or others notice; (b) alter patterns of interaction in ways that influence how people interpret and construct implications for action; and (c) influence individual and shared beliefs” (Coburn & Turner, 2011, p. 187).

## Research gap

Theoretical frameworks and empirical studies are available that address routines and their change in relation to organizational learning, improvement of teaching and learning, and data use. The studies show that routines can be deliberately changed by intentional implementation of strategies. However, in previous studies there has been no sufficient differentiation between *long-term change of routines* (referring to the ostensive aspect) and *short-term adaptation of routines* in a concrete, specific situation (referring to the performative aspect).

To change routines in the longer term (ostensive aspect of routines) an ongoing adaptation of performed situation-specific routines is required, as the ostensive and performative aspects strongly interact (Feldman & Pentland, 2003). Recently, more emphasis has been placed on the theoretical and empirical analyses of the dynamics in the formation of routines. In this way, routines are seen as being enacted through performing and patterning (Feldman, 2016; Feldman et al., 2021; Goh & Pentland, 2019). This process view leads to a better understanding of whether and how newly performed routines sustain and change routines in a longer perspective.

However, previous research lacks concepts that aid an understanding of what strategies support the situation-specific process of short-term adaptation of routines. This is problematic, for to deal successfully with a specific challenging situation the short-term adaptation of existing performed routines (performative aspect of routine) is necessary.

Furthermore, only few strategies have been identified, and a comprehensive theoretical concept of strategies for changing routines in schools is missing that conceptualizes individual and collective learning in organizations influenced by social experiences and interactions (Vygotsky, 1978) as well as by the learners’ (meta-)cognition and motivation (Winne & Hadwin, 1998; Zimmerman, 1986).

To address these gaps, we propose *focusing on short-term adaptation of existing performed routines* and extending previous theories on routines by considering theories on *self-regulated and collectively regulated learning.* Currently, theories on regulated learning (Boekaerts, 1999; Efklides, 2011; Hadwin et al., 2011; McClelland et al., 2018; Panadero, 2017; Winne & Hadwin, 1998; Zimmerman, 1986) are used not only to analyze students’ self-regulated learning but also to investigate students’ collective learning processes (Bakhtiar & Hadwin, 2020; Hadwin et al., 2011, 2018; Panadero & Järvelä, 2015). In the following, we argue that theories on regulated learning have the potential to yield a better understanding of the adapting of the routines of teachers, subgroups of teachers, and the whole school team.

To support our argumentation, we first describe the core elements of theories on regulated learning. We highlight two perspectives on regulated learning that are the most important for our theoretical framework: (a) Winne and Hadwin’s (2008) theoretical model, which views the learning process as an ‘if–then–else’ adaptation process, and (b) the concepts of co-regulation and socially shared regulation of learning (Hadwin et al., 2011, 2018; Panadero & Järvelä, 2015), as in schools not only individuals such as teachers and schools leaders but also subroups of teachers or the whole school team have to deal with challenging situations. We then show how theories on regulated learning enhance the understanding of adapting routines to a specific challenge in schools.

## Theories on regulated learning

Of the many definitions in the literature, Zimmerman’s (1986, p. 308) widely accepted definition describes self-regulated students “as metacognitively, motivationally, and behaviorally active participants in their own learning process.” Across many theories (Boekaerts, 1996, 1997; Efklides, 2011; Pintrich, 2000; Schmitz et al., 2011), the regulation of learning is understood as a cyclic and dynamic process that is divided into several phases (Panadero, 2017). The phases are not fixed but are weakly sequenced, meaning that the stages do not necessarily unfold in order (Winne & Hadwin, 1998). Several core elements of current theories can be identified that can be applied to individual learners and also to groups of learners: selection of appropriate goals, purposeful use of regulation strategies, implementation of a feedback loop during learning, and embeddedness of learning in the social context (Efklides, 2011; Hadwin et al., 2011; Panadero, 2017; Panadero & Järvelä, 2015).

### Core elements of regulated learning

The *first core element* is selection of *appropriate goals*. Individual learners or group of learners selecting appropriate goals have to take into account the conditions of the task and the individual’s and group’s conditions, such as their motivation, their knowledge in the domain, and their standards or the standards set by external authorities.

The *second core element* is *purposeful use of regulation strategies*. To reach the goals set, individual and groups of learners have to apply learning strategies. Weinstein and Mayer (1986) differentiated cognitive, metacognitive, and affective strategies:

- *Cognitive strategies* help learners and teams to pay attention to important aspects and make sure that the material is transferred into working memory for further study. They include *rehearsal strategies*, such as copying the material and underlying the important parts of the material, o*rganizational strategies*, such as outlining or creating a hierarchy to select information and to construct relations among ideas, and e*laboration strategies*, such as summarizing, creating analogies, grouping material into categories or classes, generative notetaking, or answering questions.
- *Metacognitive strategies* help learners and teams to assess the degree to which the goals set are being met and, if necessary, to modify the strategies being used to meet the goals. This includes planning the learning process, checking during the learning process for comprehensive failures and whether there is a discrepancy between the observed and the planned learning activities, and finally, evaluating the weaknesses and strengths of the applied learning process retrospectively.
- *Affective strategies* help learners and teams to focus attention; establish, maintain, and increase motivation; overcome test anxiety; and manage time effectively.

The *third core element* is *implementation of a feedback loop during learning* that relates to the planned goals and to the standards to be achieved. This includes a reflecting process of identification, analysis, and adaptation during the learning process. Feedback loops aid identification and analysis of discrepancies between planning, the implemented strategies, and actions, goals and standards, and results. The identification of discrepancies leads—ideally—to adaptation of the applied processes, motivations, beliefs, knowledge, task conditions, and tactics (Winne & Hadwin, 1998, 2008).

According to Winne and Hadwin (2008), this feedback loop process can be described as an ‘if–then–else’ process (Winne & Hadwin, 2008, pp. 303–304). Here, ‘if’ refers to the learning task, and ‘then’ refers to collections of operations (e.g., tactics and strategies) judged to be appropriate for reaching the goals of the learning task. Referring to the learning processes of teachers or school teams, the ‘if’ could be a goal the teachers and school teams have to achieve (e.g., implementation of a new diagnostic instrument), and the ‘then’ could be the routines that normally help teachers and school teams to reach the goals efficiently (e.g., monitoring the working process every week). The relation between ‘if’ and ‘then’ is in line with the ‘if–then rules’ in the theory of organizational routines by Becker (2004).

The last aspect of Winne and Hadwin’s (2008) ‘if–then–else’ process is ‘else.’ It is closely related to the evaluation of learning products and refers to another collection of operations that might be tried if the first set does not meet the goals and standards. Referring again to the school context, the ‘else’ could be teachers’ or school teams’ modified routines that have to be implemented in order to better reach the goals and standards (e.g., monitoring process with the help of external support). Winne and Hadwin (2008, p. 304) argued that particularly the switch from a ‘then’ to an ‘else’ makes a learning process a regulated learning process. This change from ‘then’ to ‘else’ is analogue to repairing routines (Feldman, 2000) as a response to performed routines not achieving the planned goals. However, Winne and Hadwin’s (1998, 2008) theoretical model allows a more fine-grained examination of this adaptation process. It is able to highlight relevant aspects that need to be regulated and adapted in order to achieve the goals set. These aspects are:

- the *conditions of individuals and groups of learners*, e.g., their motivations, domain knowledge, strategy knowledge, beliefs.
- *the task conditions*, e.g., resources and information available (such as time or definite instructions), social context.
- the *operations* applied, e.g., cognitive strategies and tactics, such as rehearsing, summarizing, outlining, searching, assembling.
- the *standards* applied, meaning the criteria to evaluate the result.

This regulation process with the aim of adapting conditions, operations, and standards to the requirements of the learning tasks in order to achieve the goals successfully is exercised by applying cognitive, metacognitive, and motivational regulation strategies (Weinstein & Mayer, 1986).

The *fourth core element* of current theories on regulated learning is that *learning is strongly embedded in the social context* and *that not only individuals but also groups regulate their learning as a collective effort* (Bakhtiar & Hadwin, 2020; Hadwin et al., 2011, 2018; Panadero & Järvelä, 2015). Although in the literature many terminologies are used to describe processes of joint (meta)cognition, motivation, emotion, and behavior regulation, a literature review by Panadero and Järvelä (2015) identified some overlaps between the concepts. In an important contribution, Hadwin et al. (2011) not only differentiated between co-regulation of learning and socially shared regulation of learning but also showed that these levels are related to each other, and that the roles of self-regulated learning of individuals, co-regulation of learning, and socially shared regulation of learning co-emerge, build into each other and mutually reinforce one another (Bakhtiar & Hadwin, 2020; Panadero & Järvelä, 2015). Furthermore, Hadwin et al., (2011, 2018) argued that self-regulation, co-regulation, and shared regulation are driven by the same cognitive architecture as described by Winne and Hadwin (1998).

More concretely, *co-regulation of learning* is defined as the temporary coordination of self-regulation between self and others and consists of “emergent interactions that temporarily mediate regulatory work (strategies, monitoring, evaluation, goal setting, and motivation)” (Hadwin et al., 2011, p. 68). A shared outcome is not the focus of the group, however. An important mechanism for learning in the group is internalization of self-regulatory processes. This is done by jointly negotiating as the group participants interact and mediate each other’s metacognitive and cognitive actions (e.g., by recommending specific tactics to address a specific goal). Importantly, the group participants bring different kinds of self-regulatory challenges and expertise to the emergent regulation, which helps to increase individual self-regulated learning competencies to achieve the (individual) learning goal. An example of co-regulation of learning is when teachers receive advice on how to improve their feedback behavior from fellow teachers who have observed them in the classroom. Their colleagues’ observations encourage them to rethink and adapt their routines regarding giving feedback to students.

*Socially shared regulation of learning* is understood as collectively shared regulatory processes, beliefs, and knowledge orchestrated in the service of a co-constructed or shared outcome. This means co-constructing shared task representations and shared goals, regulating learning through metacognitive monitoring and control of motivation, cognition, and behavior. “The ultimate goal of socially shared regulation of learning is for multiple individually-regulating individuals to co-construct and synthesize strategies, monitoring, evaluation, goal setting, planning, and shared beliefs toward shared outcomes” (Hadwin et al., 2011, pp. 69–70). Accordingly, Panadero and Järvelä (2015, p. 193) pointed to the two most salient features of socially shared regulation of learning: (a) joint cognitive and metacognitive regulatory strategies (e.g., jointly planning, monitoring, and adapting the group’s performance), and (b) group motivational efforts and emotion regulation (e.g., by social reinforcement in order to enhance the interactions between the group members). Coming back to our feedback behavior example, the same initial situation in the context of socially shared regulation of learning would probably be that if non-optimal feedback behavior in a classroom is observed, the topic will be brought to the school team. The aim would be to reflect upon on the feedback behavior of all teachers and to jointly seek strategies to develop it further.

Empirical studies have found that these core elements of regulated learning—selection of goals, use of regulation strategies, implementation of a feedback loop, and the social context—are closely linked (e.g., Efklides, 2011) and that a higher level of regulation is directly and indirectly related to a higher level of achievement (e.g., Dent & Koenka, 2016). Further, many studies revealed significant differences between learners in the quality of applying regulated learning (e.g., Edossa et al., 2019). Problems occur particularly if the strategies are insufficiently produced (production deficit) or if strategies are not used appropriately in the specific situation (usage deficit) (Lehmann & Hasselhorn, 2009). It therefore matters how well appropriate strategies are produced and selected in relation to a specific situation, task, and goal (e.g., Edossa et al., 2019; Efklides, 2011; Soodla et al., 2017). Learners and teams must know what strategies are the most effective under what conditions. Additionally, if the goals are not achieved sufficiently, learners and teams have to be able to identify, analyze, and adapt important aspects of the learning process.

### How theories on regulated learning enhance the understanding of adapting routines to the specific challenging situation in schools

Theories on routines in organizations and theories on regulated learning have several features in common. Both theories see actors as active and capable learners aiming to improve their competencies. The theories seek to explain differences between learners or teams when they are dealing with challenging tasks, goals, and situations. Moreover, they describe learning processes as goal oriented, explicit, and strategic and differentiate between individual and collective learning. Finally, both theories consider reflection and metacognition to be important for improvement.

However, theories on regulated learning have the potential to significantly extend previous theories of adapting routines in schools, particularly by describing and analyzing situation-specific learning processes much more explicitly and by focusing on the individual and collective adaptation process and the actors’ way of thinking (Earl & Louis, 2013; Schildkamp et al., 2016). The significant add-on is that through additionally considering self-regulated and collectively regulated learning theories, it becomes explicitly possible to investigate the cognitive, metacognitive, and motivational regulation strategies applied (or not applied) during the situation-specific process of adapting routines in schools. These strategies have not yet been studied comprehensively. Without targeting identification and analysis of the quality of strategy production and usage (Lehmann & Hasselhorn, 2009), it can only be ascertained that teachers or schools fail at deriving useful implications for teachers’ and students’ learning from the information available but not *why* they fail. However, by extending previous theories on adapting routines by considering theories on self-regulated and collectively regulated learning, important aspects can be analyzed that have not been considered sufficiently up to now. Some of these aspects are:

- *Identifying routines as being dysfunctional*: When, why, and in what situations are routines identified as being dysfunctional due to a mismatch between ‘*if–then*’ (Becker, 2004; Winne & Hadwin, 2008), as needing to be “repaired” (Feldman, 2000, pp. 621ff), or as needing to be resolved and mitigated (Rice & Cooper, 2010)?
- *Impulse for the adaptation of routines*: When and at what points do individuals and groups of learners decide that routines need to be adapted to a specific situation (‘*then–else*’; (Winne & Hadwin, 2008))?
- *Implemented regulation strategies*: Whether and with what quality are regulation strategies employed? What information and what data are gathered by the teachers and school leaders, the subgroups of teachers, or the whole school team to gain an understanding of the challenging situation; how are the findings evaluated and weighted? What metacognitive processes are implemented to reflect upon why any unfavorable regulation strategies were implemented? Or how can teachers or subgroup of teachers be motivated to adapt their routines to the need of a particular challenge?
- *Social setting of the adaptation process*: Who, and in what social constellation, is involved in what processes, and under what conditions is a shift made (for example, from an individual teacher to a team or to the school leader) to better deal with a specific challenging situation?

In line with research on students’ self-regulated learning (Panadero, 2017) and groups’ collectively or shared regulated learning (Bakhtiar & Hadwin, 2020; Panadero & Järvelä, 2015) it can be expected that teachers and school teams, as individuals or groups, with higher competency in regulated learning are more likely to be able to successfully adapt routines to increase the fit with the requirements and goals of a specific challenging situation. Particularly, they have a higher awareness and competency to understand whether or not motivation, knowledge, or beliefs will tend to deal successfully with the challenging situation, whether or not the standards set are adequate and in line with the competencies and motivations of the teachers and school teams, and whether or not the operations to adapt the performed routines are implemented effectively.

In the following section, we describe our newly developed theoretical model of dealing with challenging situations in schools as an interplay between routines and regulation processes, performed by teachers and school leaders, subgroups of teachers, and whole school teams.

## Adapting routines in schools by self-regulated and collectively regulated learning: A newly developed theoretical framework

**Fig. 1:**
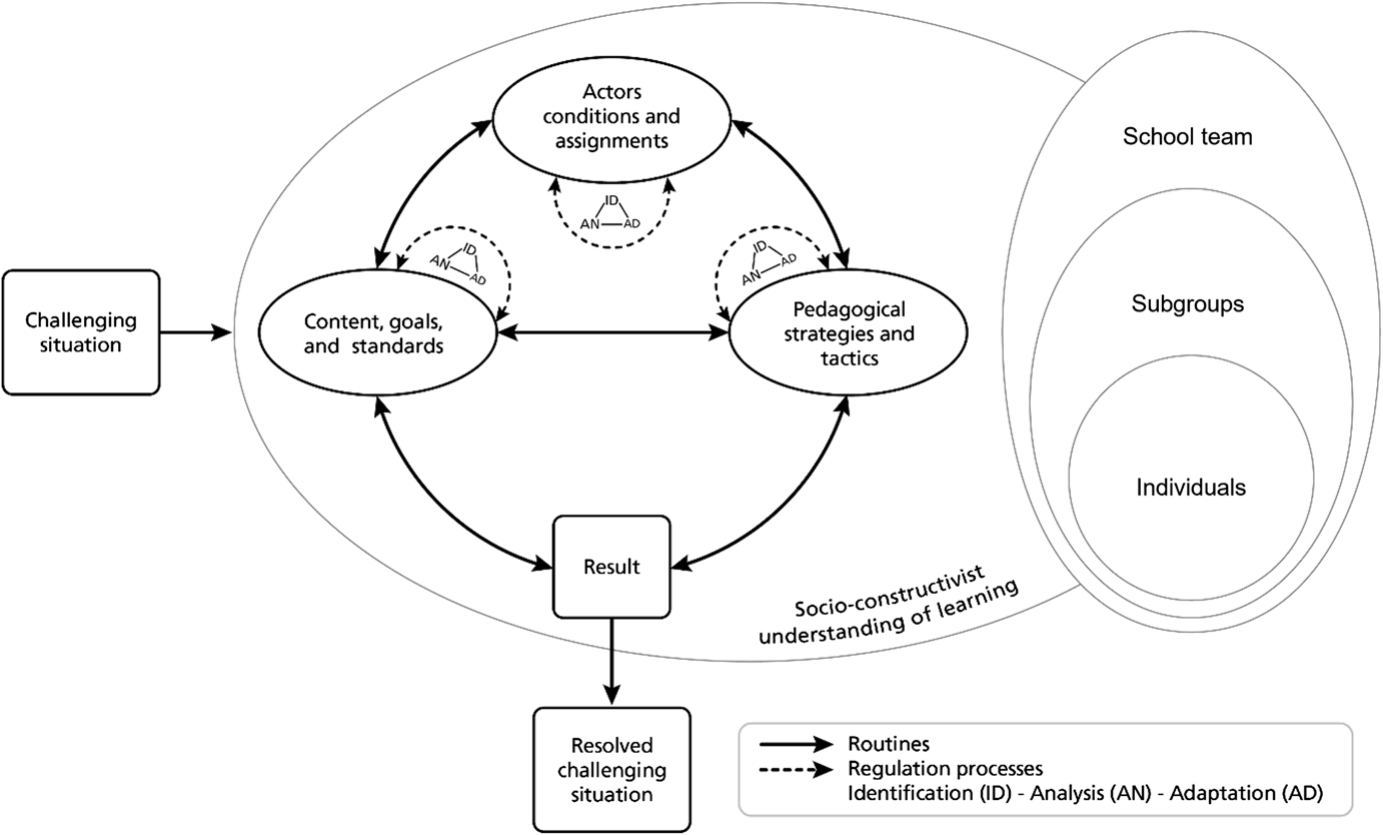
Theoretical framework of dealing with concrete challenging situations in schools as an interplay between routines and regulation processes

We describe a framework of dealing with challenging situations (see Fig. 1) as a recursive and weakly sequenced process. In this process, individuals (e.g., teachers, school leaders), subgroups of teachers, or the whole school team *enact routines* (dark arrows in the figure) (e.g., Coburn & Turner, 2011; Feldman, 2000) and *adapt routines using regulation strategies* through a process of identification, analysis, and adaptation (dotted arrows in the figure) (Panadero, 2017; Winne & Hadwin, 1998). The fundament of these processes is a socio-constructivist understanding of learning. In this approach, learning does not take place exclusively through the experiences of the individual in the objective environment but primarily through social experiences and interactions (Vygotsky, 1978).

The starting point is a *challenging situation* faced in everyday individual (i.e., teacher, school leader) or collective practice (i.e., subgroup of teachers or an entire school team). In general, we consider a challenging situation to be a demanding situation that is usually multidimensional, rather imprecise, and complex in terms of objectives and results as well as in terms of how to achieve these objectives and results. Challenging situations can occur at different levels and with different scopes and need for action: at the international level, such as the COVID-19 pandemic; at the national level, such as the introduction of national educational standards; at the regional level, such as findings of school-external evaluations; at the school level, such as the results of a parent survey or standardized achievement tests; or at the classroom level, when challenges arise in dealing with diversity or classroom disturbances.

In the following, we present our framework by focusing on routines implemented by teachers and school leaders, subgroups of teachers, or the whole school team that help them deal efficiently with these challenges and on their adaptation of performed routines in a specific situation by applying regulation strategies. Although we describe the different aspects and actions in a linear way, the process is recursive and only weakly sequenced (Winne & Hadwin, 1998). Moreover, the rationality of this learning process is limited in that individual and collective sensemaking processes (Weick, 1995) play a crucial role in identifying, analyzing, and adapting the performed routines (see also Rice & Cooper, 2010). We illustrate these processes taking the example of a challenge that many schools face: classroom disturbances (Thommen & Wettstein, 2007).

### Routines in schools

#### Understanding the content of the challenging situation, and setting appropriate goals and standards

Teachers and school teams must become clear about the challenging situation and set the goals and standards that they want to achieve when dealing with it. For this, they need to gain an understanding of the situation to be dealt with through a process of sensemaking (Coburn & Turner, 2011; Schildkamp, 2019; Weick, 1995), for example regarding the complexity of the task or the time available (Winne & Hadwin, 1998).

For instance, individual teachers might have a routine for gaining an understanding of the classroom disturbances and might try to reflect upon the problem by themselves. In contrast, at some schools there might be a routine for discussing classroom disturbances in the teacher team or the whole school team. In this process, they make collective decisions concerning the goals and standards to be set in relation to classroom disturbances. For instance, they could decide that no disturbances should be accepted in the classroom or that a low level of disturbance is constitutive for learning in a group of students.

#### At what level the challenging situation is solved, and who is involved in dealing with the challenging situation?

Dealing with challenging situation is possible on different levels: the individual level (teacher, school leader), the level of a subgroup of teachers, or the organizational level of the whole school. In some schools, there might be a routine of understanding classroom disturbances as the individual teacher’s problem. In this case, individual teachers deal with the challenging situation because they are seen as the actor who can best solve the problem. However, in other schools, classroom disturbances might be interpreted as a collective problem, as the students are taught by several teachers in the whole school. In this perspective, classroom disturbances will be solved by taking a collective perspective, and the central actor is the teaching team or the whole school (Bryant, 2018; Leithwood et al., 2020).

Further, the handling of the challenging situation is dependent on the professional competencies of the actors, including their content knowledge, metacognitive knowledge of regulation processes (Winne & Hadwin, 1998), or motivations, beliefs, and social interactions (Coburn & Turner, 2011). Accordingly, there might be a routine in the school regarding who is normally contacted (formally or informally) if problems in the classroom such as disturbances arise, for instance the most experienced teacher or a middle leader (Bryant et al., 2020). Additionally, there might be a rule concerning who *must be contacted* if classroom disturbances occur, for instance the school leader.

#### Application of pedagogical strategies and tactics to deal efficiently with the challenging situation

School leaders, teachers, subgroups of teachers, or the whole school team select from a repertoire of strategies that seem the most promising for dealing with the situation. There may be existing routines (‘if–then rules’(Becker, 2004)) regarding the choice of pedagogical strategies and also regarding the use of collecting data or strategies to reflect and monitor the process of dealing with the challenging situation (Winne, 2011).

Returning to our example, teachers or school teams might have different pedagogical strategies at hand for dealing with classroom disturbances, such as issuing warnings or moving children to different seats or classes. Additionally, teachers or school teams that are experienced in regulated learning may be in the habit of asking colleagues for feedback to help them better understand and handle the challenging situation. They may try to structure the new information, e.g., other teachers’ different ways of dealing with classroom disturbances, and analyze it efficiently, which in turn helps them to modify their pedagogical practice.

#### Analysis of the result

Ideally, the routines implemented to deal with the challenging situation will lead to a reduction in classroom disturbances and the achievement of the objectives leads to the concluding of the process.

### Regulation of routines

However, it can also happen that despite the routines implemented, no positive outcome results. The performed routines failed to achieve the goals in this specific situation. According to Winne and Hadwin (2008), when intended outcomes are not achieved, specific regulation processes are necessary, particularly the switch from ‘then’ to ‘else.’ During this regulation process, three steps are essential: First, teachers, school leaders, subgroups of teachers, or school teams have to *identify* the challenging situation and the implemented routines; second, they have to *analyze* them to find out what is not working; and third, they have to *adapt* the current routine, particularly the content, goals, and standards of the challenging situation, the actors’ conditions and assignments, and the pedagogical strategies and tactics to deal with the challenging situation. In this adaptation process, cognitive, metacognitive, and motivational regulation strategies are important. In the following three sections, this adaptation process is substantiated by referring again to our example of classroom disturbances.

#### Regulation of the content, goals, and standards

In our example, identification and analysis of the *content of the classroom disturbances as the task to deal with* are crucial for solving the situation adequately. Tasks vary in terms of complexity, the resources that are available, the time needed, or how well-defined the situation is. For regulation to take place, in-depth identification and analysis of the complex task ‘classroom disturbances’ are needed. As a regulation strategy, teachers or school teams may look for more information on the classroom disturbances by asking students for feedback or by asking colleagues to sit in on the classes to closely observe teacher-student interactions. Through this, teachers and school teams might discover that it is not individual children who are disrupting the classroom but rather that the interaction between teacher and students is disrupting teaching–learning processes (Thommen & Wettstein, 2007).

Further, teachers or school teams might realize that the *goals and standards set* are not appropriate. Accordingly, by *reflecting upon the current situation*, teachers could decide to regulate the standards and goals by changing them. For instance, teachers or school teams could reflect upon expectations regarding disturbances and clearly define what expectations should prevail in what situations.

#### Regulation of the actors’ conditions (cognition, motivation) and assignments

Based on the analysis, teachers or school teams might realize that they lack important knowledge on how to find strategies to deal more effectively with classroom disturbances. Bringing in experts, reading specialist literature, or conducting a training course could help them develop appropriate strategies and adapt their current routines.

Additionally, teachers and school teams might realize that their motivations and beliefs about handling classroom disturbances are counterproductive and that their motivational orientations or self-efficacy have to be changed (Berger & Karabenick, 2011; Efklides, 2011; Winne & Hadwin, 2008; Zimmerman & Moylan, 2009). This could be achieved by implementing motivational regulation strategies, such as calling attention to the fact that for student’s learning and well-being, it is very important to create a positive affective and cognitive climate (Efklides, 2011; Pekrun et al., 2018).

However, for successful dealing with classroom disturbances, it might be even more effective to bring in another teacher or to involve the whole school team rather than focus on only a single teacher (Coburn & Turner, 2011; Conley & Enomoto, 2005). Accordingly, actors’ assignments have to be changed.

#### Regulation of pedagogical strategies and tactics to deal efficiently with the challenging situation

Finally, one important way to adapt current routines is to regulate the strategies and tactics applied to deal with the challenging situation. Up to now, the teachers or school teams may have focused on the behavior of individual students and tried to apply social pressure as a strategy. Seeing classroom disturbances as more complex and as involving interactions in the classroom in general (Thommen & Wettstein, 2007), the teacher or school team could now focus more on a differentiated perception of disturbances, good relationships in the classroom, classroom management, and planning lessons that are stimulating and disruption-preventive.

Further, to effectively adapt the routines, it is essential to reflect upon the applied cognitive, metacognitive, and motivational regulation strategies (Weinstein & Mayer, 1986). Regulating these strategies means regulating the adaption process itself. For example, the regulation of teachers’ and school teams’ previous *cognitive strategies* could help make the challenging situation clearer. Teachers and school teams have to analyze how they located and assessed data on the situation, whether they overlooked important information (Rerup, 2009), how they made sense of the data (Schildkamp, 2019) and then sought and structured the respective new knowledge on that basis, or how they related new to previous knowledge (Coburn & Turner, 2011; Conley & Enomoto, 2005). Accordingly, production and usage of the strategies have to be analyzed in depth and then adapted based on the results of the analysis (Lehmann & Hasselhorn, 2009).

However, classroom disturbances might have a long history in a class, and the teachers or school teams may be tired of still having to deal with the same challenges. Therefore, strategies previously used to regulate *their motivations and emotions* might have to be changed by increasing the quality and adequacy of the applied strategies (Efklides, 2011; Pekrun et al., 2018).

Finally, the *metacognitive monitoring process* itself might have to be adapted, as only very informal reflection processes might have been implemented in the school and the teachers and school leaders may realize that they have to modify the applied strategies by increasing the depth of their analyzes to better deal with classroom disturbances.

## Further research

This paper presents a theoretical approach for analyzing the processes of adapting routines in order to increase the fit between the performed routine and the challenging situation that has to be dealt with in order to achieve the goals. It is a complex, iterative, and recursive process during which strengths and weaknesses of routines have to be identified, analyzed, and adapted to improve the quality of teaching and learning. To better understand this process, we propose extending previous theoretical approaches on routines by considering theories of regulated learning. The extensive body of research on self-regulated and collective learning processes (Dent & Koenka, 2016; Hadwin et al., 2011; Panadero, 2017; Panadero & Järvelä, 2015) can be used for more detailed identification, analysis, and adaptation of routines—all with a focus on improving a school’s educational practice. By focusing on teachers’ and school teams cognitive, metacognitive, and motivational regulation strategies in the process of adapting routines, it is possible to investigate research questions that address important aspects that up to now have been little studied in research on routines but also in research on educational change.

A first question to study will be how teachers and school leaders, subgroups of teachers, or the whole school team implement regulation strategies in order to adapt routines to deal more effectively with a given challenging situation, what the differences between self-regulation and shared regulation processes in the context of educational change are, and how interactions and dynamics between the members of the groups influence the shared regulation (Hadwin et al., 2011; Panadero & Järvelä, 2015).

A second research question to examine will be what difficulties teachers, school leaders, subgroups of teachers, and school teams face in order to deal effectively with current challenges in schools. According to the framework presented here, difficulties can arise in at least five areas: (1) insufficient routines when dealing with challenges in schools [‘if–then’ (Becker, 2004; Winne & Hadwin, 2008)], (2) insufficient ability to recognize when routines have become dysfunctional and must be adapted [‘then–else’ (Winne & Hadwin, 2008)], (3) insufficient ability to competently implement regulation processes for adapting necessary processes [‘else’ (Winne & Hadwin, 2008)], (4) insufficient ability to reflect upon why any unfavorable regulation strategies were implemented, and (5) insufficient reflection on the social constellation to deal with the challenging situation, considering also the micropolitics in schools and the respective dynamics between teachers, school leaders, and school authorities in terms of hierarchy, control, power, and conflicts (Altrichter & Moosbrugger, 2015). Knowing these difficulties and what difficulty in a specific school is predominant might aid development of more precisely custom-fit intervention programs that extend existing intervention programs for educational change and the improvement of schools (Creemers & Kyriakides, 2012; Kyriakides et al., 2019; Vanhoof et al., 2013).

Third, considering current discussions on the task- and domain-specificity of regulated learning (e.g., Fitzgerald et al., 2017; Meijer et al., 2006; Puranikas et al., 2019), future studies could examine whether regulation processes to adapt performed routines in schools differ in terms of the content of the routines (e.g., routines in monitoring teaching and learning vs. routines in monitoring teachers’ collaboration) or in terms of the challenging situation that teachers and school leaders have to deal with (e.g., dealing with socially disadvantaged students vs. motivational problems of teachers).

Fourth, analyzes are needed to find out according to what criteria it can be shown that school actors have implemented goal-appropriate routines and regulation processes, what role teachers, subgroups of teachers, and the whole school team take in this process, and how these processes on the individual, interpersonal, and organizational levels are interrelated.

Fifth, it has to be analyzed whether higher quality of these regulation processes indeed leads to a better result when dealing with challenges in schools, as research on the effects of students’ self-regulated learning on achievement shows (Panadero, 2017) and whether schools with higher competency in self-regulated and collectively regulated learning also have a greater capacity to improve schools (Hallinger & Heck, 2010; Mitchell & Sackney, 2011; Sleegers et al., 2014).

Sixth, investigations are needed on how regulation strategies might be fostered in order to increase teachers’ and school leaders’ capacity for developing and adapting routines and educational change (Mitchell & Sackney, 2011).

Seventh, it has to be analyzed whether, when, and how the adaptation of situation-specific performed routines (performative aspect of routines) influences also the ostensive aspect of routines (Feldman, 2003) and how the performing and patterning of the identification, analyzes, and adaptation processes influence the change of routines in schools in a longer term (Feldman et al., 2021; Goh & Pentland, 2019).

And finally, we need to keep in mind that studying regulated learning processes empirically is challenging (Wirth & Leutner, 2008). Extensive discussion of methodological issues has revealed that the usual standardized instruments are only partly functional for capturing regulation processes. Accordingly, future research has to investigate what methodology and methods can best capture regulation strategies. Instruments are needed that measure school actors’ day-to-day practice as accurately as possible (Camburn et al., 2010). In particular, there must be a focus on capturing the quality and not only the quantity of the implemented regulation strategies (Wirth & Leutner, 2008).

Taking together, investigating routines in schools theoretically and empirically with a focus on the concepts of self-regulated and collectively regulated learning has great potential in research on adapting routines not only from a research perspective but also regarding real-world practice. Although this approach certainly poses challenges, it may help make it possible to better understand, evaluate, shape, support, and adapt routines in practice and to support teachers and school leaders in developing capacities for sustainable change.
